# Behavioral Capital Theory via Canonical Quantization

**DOI:** 10.3390/e24101497

**Published:** 2022-10-20

**Authors:** Raymond J. Hawkins, Joseph L. D’Anna

**Affiliations:** 1 Economics Department, University of California, Berkeley, CA 94720, USA; 2 Wyant College of Optical Sciences, University of Arizona, Tucson, AZ 85721, USA; 3 Zeconomy Inc., New York, NY 10023, USA

**Keywords:** capital theory, quantum cognition, behavioral economics

## Abstract

We show how a behavioral form of capital theory can be derived using canonical quantization. In particular, we introduce quantum cognition into capital theory by applying Dirac’s canonical quantization approach to Weitzman’s Hamiltonian formulation of capital theory, the justification for the use of quantum cognition being the incompatibility of questions encountered in the investment decision-making process. We illustrate the utility of this approach by deriving the capital-investment commutator for a canonical dynamic investment problem.

## 1. Introduction

Capital theory is the area of economics that addresses the intertemporal allocation of resources by rational economic agents [1]. Time is of importance because the agent must decide how much current output to divert from current consumption to create the capital goods required for future production. The agent is assumed to make decisions in a rational manner, where to be rational is to maximize the utility of current and future consumption in general and to maximize the present value of profit if the agent is a producer in particular. And while rationality has been extended to include some behavioral considerations, in these analyses, all questions encountered by an agent in the temporal allocation of capital are assumed to be compatible: either simultaneously answerable or, when answered sequentially, the order of the questions does not matter.

In this paper, we build on the literature that, having found these assumptions wanting, restructured decision theory in a more psychologically sound manner [2,3,4,5,6,7,8,9,10,11]. This literature provides a formalism for representing the indeterminacy that accompanies decision making and the reality that most questions encountered by an agent in capital theory are incompatible: they either cannot be answered simultaneously or, when answered sequentially, the order of asking matters. We find in Section 2 that enhanced psychological realism in decision theory and indeterminacy recommend a move from set-theoretic probability to probability based on projective geometry, a move that allows us to leverage the considerable development of this form of probability in the field of quantum theory. Inspired by the usefulness of quantum theory methods in decision theory, we show there is a natural justification for its use in capital theory: first by reviewing, in Section 3, the traditional formalism of capital theory and its “Hamiltonian economics” (i.e., classical mechanics) representation advocated by Weitzman [12], and then in Section 4 by observing that the need to use the formalism of quantum theory for decision theory in capital theory recommends the use of canonical quantization to extend Weitzman’s Hamiltonian economics in a psychologically coherent manner. We close in Section 5 with a discussion and summary.

## 2. Decision Making and Quantum Cognition

Time commonly enters economics in the form of the discount factor; the coefficient that, for each point in the future, indicates how much less utility at that point in time is valued today [13]. The shape of the discount factor as a function of horizon was interpreted by Phelps and Pollak [14] as reflecting a psychology of decision making that involved a continuum of decision makers—the current self and all future selves—in the determination of the discount factor, a construct further elaborated by Laibson [15] in their interpretation of the future decisions one makes as being made by different selves. The subsequent game-theoretic literature that grew from the work of Phelps and Pollak was advanced considerably by Lambert-Mogiliansky and colleagues [3,4,5] who realized that the questions entertained by these selves are, in general, incompatible. From a psychological perspective, this work took seriously the observation of Tversky and Simonson [16] that

“[t]here is a growing body of evidence that supports an alternative conception according to which preferences are often constructed—not merely revealed—in the elicitation process. These constructions are contingent on the framing of the problem, the method of elicitation, and the context of the choice.”

and of Ariely, Prelec, and Lowenstein [17] that

“valuations are initially malleable but become ‘imprinted’ after the agent is called upon to make an initial decision.”

The notion that preferences are constructed and that, prior to construction, an agent resides in an indeterminate preference state differs materially from the traditional perspective in economics that an agent is endowed with a preference set.

The existence of an indeterminate preference state renders classical, set-theoretic probability inadequate for analyzing expected outcomes in decision theory. This is the basis of considerable literature on the use of projective geometry-based probability, such as that encountered in quantum theory, which is commonly referred to as quantum cognition. General quantum cognition references include the work of Busemeyer et al. [6,7,8,18] and Khrennikov et al. [9,10,11,19]. One of the earliest works in this area is that of Aerts and Aerts [2]. A comprehensive perspective on quantum models in the social sciences is given in the handbook that is reference [20].

To illustrate the need for quantum cognition in capital theory, we consider the following adaptation/paraphrasing of the trip problem posed by Bruza et al. [8]. A key concept of quantum theory—the uncertainty principle—becomes relevant when an individual’s understanding of two events requires a change in perspective, a change that can imply incompatibility. In our capital theory setting, we consider a planned investment. This investment requires the postponement of a given amount of consumption to support the creation of some corresponding capital in the future. In the present, an agent will judge a given investment by considering their net present satisfaction with the loss of current consumption and anticipated future capital gain. In the future, the agent’s preferences may differ from those of their past self. They may, for instance, place less value on prior postponed consumption and more value on increased current capital. Because humans are present biased [13], the agent’s estimation its future self’s preferences requires a change in perspective to that of (effectively) a different person. Invariably, a different person’s preferences and the agent’s own will not be perfectly consistent. The experience of taking an inconsistent perspective will leave the agent in a state unattainable from their original perspective. By determining the investment value ascribed by their future self, they will render their own valuation uncertain. Thus, an uncertainty principle arises from the impossibility of determining the precise value of an investment simultaneously from both the perspective of an agent’s current and future selves. These present and future perspective valuations are incompatible because they cannot be jointly determined simultaneously. Though incompatible, the perspectives are complementary in the sense that one without the other does not represent the entirety of the information available. Further, the notion of superposition emerges because when an agent’s judgement of the investment is certain (e.g., good, bad, or indifferent) from its current perspective, their cognitive state must be dispersed, or indefinite, with respect to the perspective of their future self.

Bruza et al. argue that the principal of complementarity, together with the uncertainty and superposition principles—which describe key qualities of quantum systems and motivate essential differences between classical and quantum probability theory—are crucial to understanding limitations in human behavior and decision making that reside outside the bounds of rationality or what can be explained by classical cognitive theory. Thus, they motivate the application of quantum probability theory in models of cognition. Having identified these same principals at work in capital and investment decision making, our assessment is that the application of quantum probability to capital theory is similarly necessary.

Intuition concerning the failure of capital and investment to commute can be seen in a further adaptation/paraphrasing of the trip example of Bruza et al. Consider deciding whether you believe you will either be *satisfied*, *neutral*, or *dissatisfied* with an investment, and associate *A*, *B*, and *C* with the event of arriving at each corresponding answer. Let *S* represent your initial, undecided cognitive state.

Using a quantum probability approach, we can represent *S*, *A*, *B*, and *C* as unit vectors within a multi-dimensional Hilbert space that encompasses all possible beliefs based on the available information. The vectors *A*, *B*, and *C* form an orthogonal set of axes in the sub-space encompassing beliefs that have any bearing on the question. The event of answering the question is represented by the transition of the cognitive state from *S* into a single decided state among *A*, *B*, or *C*.

Considered from the perspective of your current self, the probability of event *A* (deciding you believe the investment will be satisfying) or event *B* (deciding you are neutral) is given by the projection of *S* onto a plane spanned by the vectors *A* and *B*, the squared length of the projection being the joint probability of event *A* or *B* (deciding you believe you will at least be neutral). Similarly, the probability you believe you will be dissatisfied is given by the squared length of the projection of *S* onto *C*.

To judge the satisfaction with the investment from the perspective of your future self requires a change in perspective. This change can be accomplished by rotating from the *A*, *B*, and *C* decision axes of your current self to the axes *U*, *V*, and *W* of your future self. The degree of rotation between the two perspectives is determined by the similarity between the perspectives of your current and future selves. Because, as mentioned above, people are present biased [13], a material degree of rotation is expected. The probabilities of answers from the perspective of your future self are determined by the projection of *S* onto the axes *U*, *V*, and *W*. And if, for example, you decide that your future self will be neutral with respect to the investment, then your state vector *S* will be aligned with *V* and you will become undecided about your current self’s assessment of the investment because the rays *A*, *B*, and *C* are oblique with respect to ray *V*: being certain about event *V* forces you to be superposed with respect to events *A*, *B*, and *C*. Finally, note that the result of (i) projecting *S* onto the *A*, *B* plane and projecting that result onto *V* will, in general, differ from the (ii) projecting *S* onto *V*, and then projecting the result onto the *A*, *B* plane. Because a present bias is expected to generate a rotation between the perspectives of your current and future selves, the order of the projections matters; a present bias implies that the events corresponding to the investment decision are incompatible and, therefore, do not commute.

A compelling demonstration of the fruitful application of these themes in economics can be found in the work of Lambert-Mogiliansky and colleagues [3,4,5]. They show that the multiple-self perspective and associated quantum cognition formalism provide a coherent framework for interpreting a number of observed behavioral economics phenomena. The implication for capital theory is that the intertemporal optimization of investment will generally involve an agent’s assessment of preferences from both their own and their future self’s perspectives, and because such a multi-perspective assessment is well accounted for by techniques of quantum cognition (see reference [8] and references therein), it follows that quantum cognition can be expected to be required for a psychologically sound representation of capital theory.

## 3. Capital Theory and Classical Mechanics

Given the need to incorporate quantum cognition into capital theory, a natural basis for the formal introduction of this approach is in order. Capital theory has a well-established formalism for treating investment under uncertainty [21,22,23,24,25,26], with applications ranging from Ramsey’s optimal rate of saving for a nation to Hotelling’s optimal extraction of a nonrenewable resource and Clarke’s work on the optimal management of fisheries [27,28,29,30]. And while Ramsey did use variational calculus in his pioneering work, economics more typically uses optimal control theory and dynamic programming for these problems. By contrast, Weitzman made a compelling case for Hamiltonian economics: the application of classical (or analytic) mechanics developed by Hamilton to economic control problems [12]. Specifically, Weitzman observes that

“What economists call ‘capital’ corresponds to what the physicists call a ‘generalized coordinate’, while economists’ ‘price’ of capital corresponds to their ‘generalized momentum’. The current value Hamiltonian for economists is ‘income’, while for the physicists it is ‘energy’. Otherwise, the mathematical structure of the two systems is essentially isomorphic.”

Weitzman’s work is particularly important because, in addition to furthering the reach of Hamiltonian mechanics, it provides a natural way to introduce quantum theory—the formalism needed for quantum cognition—into capital theory, the use of canonical quantization, to which we now turn.

## 4. Canonical Quantization of Capital Theory

Weitzman motivated Hamiltonian economics with the example of an entrepreneur considering the start and growth of a widget business. Widgets are assumed to have a linear demand function

(1) $$D\left(P\right)=\frac{\bar{P}-P}{b}$$

for widget price *P* where $\bar{P}\geq P$ and *b* are positive constants with an associated inverse demand function

(2) $$P\left(D\right)=\bar{P}-bD$$

and revenue function

(3) $$\Phi \left(D\right)\equiv P\left(D\right)D=\bar{P}D-bD^{2}.$$

The widgets are made by infinitely lived robots with one-time cost *c*. The interest rate is ρ. Each robot takes time τ to be adjusted when it arrives, so if *I* new robots arrive, they will require τI time for adjustment. At time *s*, there will be s/τ adjusted machines and I−s/τ machines awaiting adjustment. The lost widget production per period from the adjustment process is

(4) $$\int_{0}^{\tau I}\left(I-\frac{s}{\tau }\right)ds=\frac{1}{2}\tau I^{2}$$

where τ/2 is the cost-of-adjustment coefficient.

The entrepreneur now faces the dynamic investment problem: given *K* operating robots and *I* new robots at time *t*,

(5) $$\mathrm{maximize}\;\int_{0}^{\infty }\left[\bar{P}K-bK^{2}-cI-\frac{1}{2}\tau I^{2}\right]e^{-\rho t}dt$$

subject to

(6) $$\begin{array}{cc} \dot{K} & =I \end{array}$$

where the dot indicates differentiation with respect to time and

(7) K(0)=0.

The discount rate ρ is the rate of return associated with investments with a similar level of risk and could be expressed in terms of the capital asset pricing model by

(8) $$\rho =r_{f}+\beta \left(r_{m}-r_{f}\right)$$

where $r_{f}$ is the risk-free rate, β is the normalized covariance of the investment return with the market, and $r_{m}$ is the expected market return. Generalizing *K* and *I* to capital stock and investment, respectively, Weitzman’s example becomes a general expression of the key investment question of capital theory.

The integral that an economist would maximize—shown in Equation (5)—can be converted into an action, A, that a physicist would minimize (well, take the variation of) by substituting the constraint given by Equation (6) into Equation (5) and changing the sign of the integrand:

(9) $$A=\int_{0}^{\infty }\left[\frac{1}{2}\tau {\dot{K}}^{2}+c\dot{K}+\bar{P}K-bK^{2}\right]e^{-\rho t}dt.$$

The corresponding Lagrangian and Hamiltonian can be found by playing off an analogy with the terms that a physicist would recognize as the momentum and the potential: $\tau {\dot{K}}^{2}/2$ and $-\bar{P}K+bK^{2}$, respectively. The “H=T+V so L=T−V” shortcut to getting the Lagrangian from the Hamiltonian is thwarted, however, by the term $c\dot{K}$ which does not seem to have any natural home in either the momentum, *T*, or the potential, *V*. So, recalling that

(10) $$p=\frac{\partial L}{\partial \dot{q}}$$

and

(11) $$H=p\dot{q}-L,$$

and setting $\bar{P}=b=\rho =0$ temporarily to make the math easier, we have that

(12) $$H=\frac{1}{2}\tau {\dot{K}}^{2}+c\dot{K}=\frac{\partial L}{\partial \dot{K}}\dot{K}-L$$

so

(13) $$\dot{K}\frac{\partial L}{\partial \dot{K}}-L=\frac{1}{2}\tau {\dot{K}}^{2}+c\dot{K}.$$

The solution to

(14) $$xy^{\prime }-y=ax^{2}+bx$$

is

(15) $$y=ax^{2}+bx\ln x+c_{1}x$$

where $c_{1}$ is a constant. From this, we back out the Lagrangian

(16) $$L=\left[\frac{1}{2}\tau {\dot{K}}^{2}+c\dot{K}\ln\dot{K}+\bar{P}K-bK^{2}\right]e^{-\rho t}$$

and momentum

(17) $$p=\left[\tau \dot{K}+c\left(\ln\dot{K}+1\right)\right]e^{-\rho t},$$

from which the Hamiltonian

(18) $$H=\left[\frac{1}{2}\tau {\dot{K}}^{2}+c\dot{K}-\bar{P}K+bK^{2}\right]e^{-\rho t}$$

follows from Equation (11) and where we are interpreting $\frac{1}{2}\tau {\dot{K}}^{2}+c\dot{K}$ and $-\bar{P}K+bK^{2}$ as the economic equivalent of kinetic and potential energy, respectively.

A compelling feature of this approach to economics is that if one makes the natural choice of capital stock, *K*, as the canonical position, then the equation of motion for the capital stock is

(19) $$\dot{K}=\left\{K,H\right\}$$

where the Poisson brackets are given by

(20) $$\left\{A,B\right\}=\left(\frac{\partial A}{\partial K}\frac{\partial B}{\partial p}-\frac{\partial A}{\partial p}\frac{\partial B}{\partial K}\right)$$

for the general functions A(q,p) and B(q,p) of the canonical position and momentum, *q* and *p*, respectively. The canonical momentum *p* is defined by [31]

(21) $$p=\frac{\partial L}{\partial \dot{q}}$$

where *L* is the Lagrangian

(22) L=pq−H,

which for our system is given by Equation (16) and from which follows the canonical momentum

(23) $$p=\left[\tau \dot{K}+c\left(\ln\dot{K}+1\right)\right]e^{-\rho t}$$

with equation of motion

(24) $$\dot{p}=\left\{p,H\right\}.$$

The extension of capital theory to include the treatment of incompatible phenomena in a manner that is as formally similar as the Hamiltonian approach of Weitzman as possible can be accomplished through Dirac’s canonical quantization [32]. In this approach, the canonical position and momentum *q* and *p* are replaced by operators $\hat{q}$ and $\hat{p}$ on the Hilbert space $H=L^{2}\left(R^{2}\right)$ [33]

(25) $$\hat{q}\psi \left(K\right)=K\psi \left(K\right),$$

and

(26) $$\hat{p}\psi \left(K\right)=-i\hbar \frac{\partial }{\partial K}\psi \left(K\right),$$

where $\psi^{*}\left(K\right)\psi \left(K\right)$ is the probability of finding the system with capital stock *K*, the asterisk (*) indicates complex conjugation, and where the Poisson bracket is replaced by the commutator

(27) $$\left[\hat{q},\hat{p}\right]=i\hbar$$

with $i=\sqrt{-1}$ and with *ℏ* being an experimentally determined constant with units of income to ensure dimensional consistency.

The failure of $\hat{q}$ and $\hat{p}$ to commute shown in Equation (27) is the feature of the quantum approach that enables the treatment of investment-decision situations involving incompatible operators. For example, inverting Equation (23) and solving for $\dot{K}$ yields

(28) $$\dot{K}=\frac{c}{\tau }W\left(\frac{\tau }{c}e^{\frac{p\exp\left(\rho t\right)-c}{c}}\right)$$

where W(x) is the Lambert W-function. Transforming to operator form via Equation (26) and taking the c→0 limit for purposes of illustration, we find that

(29) $$\left[\hat{K},\hat{I}\right]=2\tau^{-1}i\hbar e^{\rho t}.$$

Capital stock and investment do not commute because they are incompatible and the degree to which they do not commute increases with the investment horizon *t*.

The exponentially growing commutator seen in Equation (29) is the “cousin” of the exponentially dying commutator seen in studies of non-conservative forces. Tartaglia [34], for example, breaking time symmetry with the factor $\exp\left(\delta t\right)$, considers the Lagrangian

(30) $$L_{1}=\frac{1}{2}m\dot{x}e^{\delta t}$$

and finds that the associated Euler–Lagrange equation of motion “reduces to the equation of motion of an object through a viscous medium generating the linear friction force $F=-m\delta \dot{x}$” (see also Kobe et al. [35]), and that the commutator between the kinetic momentum operator, ${\hat{p}}_{k}$, and the coordinate operator, $\hat{x}$, is

(31) $$\left[\hat{x},{\hat{p}}_{k}\right]=i\hbar e^{-\delta t}.$$

In this commutator, we see that it begins in the usual quantum form (i.e., =iℏ) and over time becomes classical (i.e., =0).

The exponentially varying commutators—Equations (29) and (31)—provide an answer to the question posed by Weitzman concerning the physical analogy of the discount factor $\exp\left(-\rho t\right)$:

“There are some differences, of course, between Hamiltonian physics and Hamiltonian economics. The physicist almost always works with a system where energy is conserved, which corresponds in economics to a limiting case of zero discounting—and it is not clear what is the physical significance of positive discounting (does it represent a universe whose energy is ‘leaking out’ at a constant rate ρ?). Economists are frequently interested in inequality constraints, for example the non-negativity of capital or the boundedness from below of net investment, and their prices are non-negative, whereas in physics it is as normal for position or momentum to be negative as positive. Nevertheless, what is most striking in the comparisons is the relative similarity of the underlying mathematical structure, rather than the differences.”

When ρ<0, a borrower is charging you to hold your money. This corresponds to the positive exponentials in the Lagrangian for the objects in a viscous medium or experiencing a linear frictional force discussed above. It is a dissipation of income encountered, for example, in the storage cost of commodities and the recent imposition of negative nominal interest rates by central banks. When ρ>0, a borrower is paying you to hold your money. Physically, this would correspond to an object extracting energy from a system, such as a photon amplification in a gain medium. Economically, this is the usual situation one encounters when one lends money.

## 5. Discussion and Summary

The empirical justification for the use of a quantum-theoretic probability framework in capital theory is the observation that the intertemporal optimization of capital requires knowledge of the preferences of one’s current and future selves, knowledge that can only be obtained by the measurement process of constructing the answer to the investment question. Until that measurement has happened—i.e., one has “pulled the trigger” on an investment decision, so to speak—the economic agent pondering the investment is in an indeterminate state where, for example, the binary answers of “invest” and “don’t invest” are in a superposition. Consequently, these decision are said to be incompatible—either cannot be made simultaneously or, when sequentially made, order matters—and there is growing literature demonstrating that when one encounters incompatible questions in the social sciences generally and in economics in particular, the probabilistic methods developed to deal with incompatible questions in physics (e.g., the position and momentum of a particle) are appropriate [2,3,4,5,6,7,8,9,10,11]. To this literature, we add capital theory.

An important precedent to our work is that of Contreras et al. [36,37] who made an extensive study of the relationship between optimal control theory and quantum theory. In this work, they built on their observation of the equivalence between the Pontryagin equations and those of classical constrained systems and showed that the Schröedinger equation that follows from the quantization of the classical system is dynamically equivalent to the Hamilton–Bellman–Jacobi equation. In particular, they found that the Bellman theory is equivalent to the classical (ℏ→0) limit of the quantum results in general and of Feynman’s path integral approach in particular. Our work builds on this foundation by establishing a psychological basis for the use of quantum theory in the application of the Hamiltonian dynamics known as capital theory, elaborating the use of canonical quantization as a natural way to accomplish the needed quantization, and using the connection between the results of this quantization and prior work in the quantization of dissipative systems to illuminate the interpretation of discounting in Hamiltonian economics.

**Figure 1:**
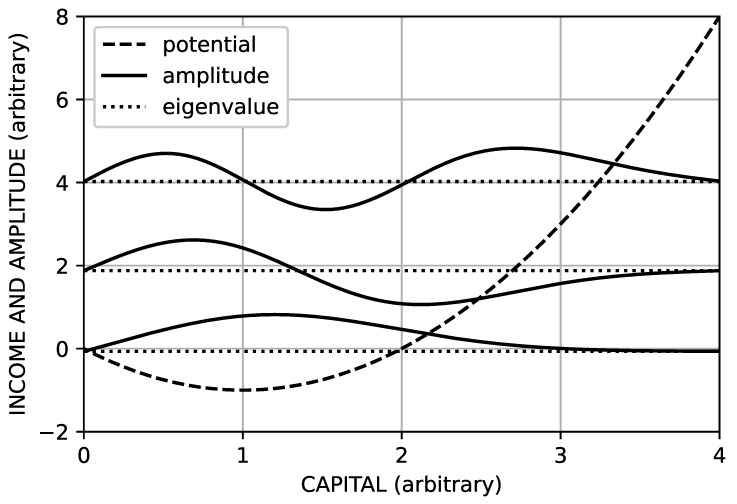
The potential function for the widget entrepreneur example as a function of the level of capital stock together with some of the associated probability amplitudes.

In Weitzman’s Hamiltonian economics, the focus is on a phase space defined by the coordinates *K* and *I*, with the evolution of the investment process being a trajectory through this phase space given by Hamilton’s equations. By contrast, the quantum approach to capital theory treats income and capital probabilistically via a Schrodinger equation, the components of which are shown in Figure 1. In this figure, we see the potential function, $V=bK^{2}-\bar{P}K$ generated with b=1 and $\bar{P}=2$, together with the lowest three of the associated amplitude solutions to the Schrodinger equation; each amplitude is graphed with respect to its associated eigenvalue. The level of capital at a given point in time is the expectation of capital with the probability given by $p\left(K\right)=\psi \left(K\right)\psi^{*}\left(K\right)$ where ψ(K) are solutions to the associated Schrodinger equation and the superscript * indicating complex conjugation. The lowest of the amplitudes with the single lobe is the one associated with the economy being in an equilibrium or steady state. The other amplitudes are associated with excitations of the economy and give rise to materially different expectations of capital, the empirical examination of which is the subject of future work. Clearly, the quantum approach to capital theory that arises as a consequence of incompatibility in investment decisions gives rise to a rich set of possible investment outcomes.

In summary, we have developed a behavioral form of capital theory based on the psychological notion that preferences are constructed in the elicitation process. This notion differs materially from the standard economic assumption that preferences are both part of one’s endowment and of a fixed temporal order. This suggests that some questions intended to reveal preferences will be incompatible, and that a probabilistic framework for handling incompatible questions is required. The probabilistic framework of quantum theory is, in some sense, the minimal deviation from the set-theoretic probability framework used for compatible questions and, thus, an appropriate choice for incompatible questions. Capital theory deals, in general, with the intertemporal optimization of capital levels and, in particular, with the withholding of capital from current consumption for the generation of future capital. While standard economic theory does recognize this situation as giving rise to conversations between current and future selves regarding the investment process, a contribution of this paper is the observation that the questions that arise in these conversations are generally incompatible. We introduced quantum cognition into capital theory by quantizing Weitzman’s Hamiltonian formulation of capital theory using the canonical quantization of Dirac. Illustrating the resulting quantized capital theory with a fundamental investment example from Weitzman, we found a relationship with dissipative quantum systems. Weitzman’s development of capital theory as Hamiltonian economics is remarkable in the breadth of the practical intertemporal optimization applications that it covers. The behavioral capital theory based on canonical quantization holds promise for the development of Hamiltonian economics with a greater psychological reality and of similar reach.
